# Upconversion-Enhanced Luminescence in PMMA Doped with Rare Earth Ions by Plasmonic Resonance with Metallic Nanoparticles

**DOI:** 10.1021/acsomega.4c07927

**Published:** 2025-03-17

**Authors:** Oswaldo Gallardo-Rivera, Anahi Rivera, Luis Octavio Meza Espinoza, Zorayda Lazcano Ortiz

**Affiliations:** † Instituto de Física Luis Rivera Terrazas, Benemérita Universidad Autónoma de Puebla, Puebla 72000, Mexico; ‡ Ingeniería Automotriz, Universidad Politécnica de Amozoc, Amozoc de Mota 72980, Puebla, Mexico

## Abstract

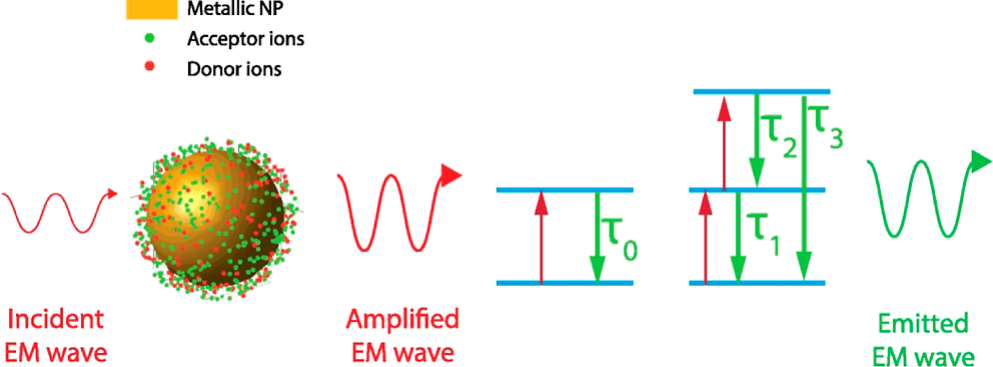

This study investigates the luminescent dynamics of poly(methyl methacrylate) (PMMA) doped with rare earth ions, focusing on donor and acceptor ions. The acceptor ions feature two excited energy levels, enabling upconversion through energy transfer (ET) with the donor ions. Additionally, this study examines how the luminescent dynamics is affected by the enhanced electric field achieved through plasmonic resonance with metallic nanoparticles (NPs). The motivation behind this study lies in the necessity to enhance the luminescence properties of materials for advanced applications in bioimaging and optical sensors. Utilizing Förster’s theory and the MNPBEM toolbox in MATLAB, the optimal NP radii for gold and silver, as well as the excitation wavelengths that maximize electric field amplification, were determined. Our findings show that silver NPs offer superior electric field enhancement (up to 8.7 times) compared to gold NPs (3.6 times). Emission amplification, influenced by the NP radius, excitation wavelength, and ion density, showed a significant correlation due to ET and excited-state absorption processes. Notably, silver NPs exhibited a maximum emission amplification of the second excited level of the acceptor ions of approximately 150 times. These findings offer valuable insights into utilizing plasmonic resonance and rare earth doping to enhance luminescent properties in materials with potential applications in biomedical imaging, biosensing, photovoltaic devices, and other advanced optical technologies. This work differs from previously published studies by focusing on the interaction of both excited-state absorption and ET in a model that considers the upconversion process and demonstrating a 2-fold higher electric field enhancement with silver NPs compared to gold. Furthermore, this study explores the optimization of NP size and excitation wavelengths to maximize the enhancement, which, to our knowledge, was not previously considered.

## Introduction

Doped systems refer to materials deliberately altered by the intentional introduction of impurities, called dopants, to modify their physical and chemical characteristics. These dopants often involve atoms of elements categorized as “rare earth”, which are distinguished by their unique magnetic, phosphorescent, and catalytic properties. For instance, neodymium is extensively employed in the creation of powerful magnets essential to computer disk drives. Similarly, cerium plays a vital role as a component in autocatalysts, and all rare earth elements contribute to the production of flat-screen TVs.^1^ Rare earth-doped materials also enable further improvement through the upconversion process, which finds applications in the bioimaging of living cells, biosensors, chemosensors, and other optical fields.^2−6^

On the other hand, poly(methyl methacrylate) (PMMA) is widely recognized for high light transmittance and chemical resistance. When this polymer is doped with rare-earth ions, some of its properties, such as electrical conductivity, photoconductivity, and magnetic characteristics, are enhanced. Furthermore, the distinctive attributes of polymers enable the production of films, coatings, and interface agents, making them ubiquitous components in numerous technological applications.^7−10^

Upconversion (UC) is an optical process that converts low-energy light into high-energy light (such as visible light) through a nonlinear process.^11^ Typically, this process is generated by two mechanisms that can occur simultaneously or individually; the mechanisms are the following: (1) upconversion by excited-state absorption (UC-ESA) and (2) upconversion by energy transfer (UC-ET).^12^

The UC-ESA mechanism is as follows: first an optically active ion in the ground state is excited by a photon that is resonant with a higher level, and then an electron is promoted to its first excited state. Subsequently, a second photon hits the excited ion, promoting the excited electron to a second-energy state. Once the ion has received the energy of two photons, it can emit a photon with twice the energy. The second process of UC-ET is the following: an ion known as the “donor” absorbs the energy from an incident photon. Subsequently, the donor transfers this energy to an ion in the ground state known as the acceptor. After this, another surrounding donor ion that is in its excited state transfers its energy to the same acceptor that is now excited, promoting it to a higher state. Finally, the acceptor can emit a photon with more energy than the incident photon.

The amplification of luminescence in doped materials using gold and silver nanoparticles (NPs) has been studied;^13,14^ however, the UC process was not considered. The inclusion of metallic nanoparticles (MNPs) is because they can act as amplifiers of the incident electromagnetic field when excited at their plasmonic resonance frequency, improving ET efficiency from the excited states of the doped material.^15,16^

Plasmons are collective oscillations of free electrons in a metal; these oscillations occur at a well-defined frequency called “plasmon frequency”. For MNPs, which have a size comparable to the depth of the metal skin, the electric field of incident light is able to penetrate the metal and polarize the conduction electrons.^17^ One of the effects of these collective oscillations is the significant increase in absorption and scattering cross-section as well as the amplification of the local optical electromagnetic field.^18^ When rare earth ions are close to a metallic nanostructure, the enhanced electromagnetic field generated by surface plasmonic resonance can affect light emission and amplify it. The enhancement of luminescence can also be achieved through other processes; for example, the use of micro- or nanostructured substrates has been reported, where the improvement is not attributed to plasmonic effects.^19,20^ Additionally, microfabricated chips utilizing a hybrid platform that combines silver NPs and diphenylalanine nanotubes have been investigated, where fluorescence enhancement is achieved through electro-optical synergy.^21^ In this latter work, a plasmonic response is leveraged, but the upconversion process is not considered.

The applications of upconversion and plasmonic resonance in gold and silver NPs doped with rare earth elements span a wide range of technological and biomedical fields. An upconversion-enabled seedless photochemical approach has been demonstrated to synthesize silver nanostructures, highlighting its potential in microlasers, biosensors, antibacterial applications, and catalysis.^22^ Previous work reviewed the latest advances in rare earth-doped upconversion nanoparticles (UCNPs), emphasizing their use in latent fingerprint detection, drug delivery, and anticounterfeiting.^23^ In biological applications, UCNPs are widely used due to their enhanced stability against photodegradation and greater tissue penetration, making them suitable for fluorescence imaging, biolabeling, and cancer therapy.^24^ Another work provided a review of the synthesis methods and optical sensing applications of plasmonic metal NPs, including gold and silver, emphasizing their role in pathogen detection and cancer diagnostics.^25^ These studies highlight significant applications and advancements in enhancing the efficiency and functionality of various technological and biomedical devices.

To simulate the effect of MNPs on the emission of rare earth ions incorporated in PMMA, a model based on the Förster theory is applied, integrating the MNPBEM tool in MATLAB.^26^ MNPBEM is an open-source toolbox that solves Maxwell’s equations using the boundary element method (BEM). This tool was used to simulate the effects of MNPs on the electromagnetic field. The formal structure of this method is presented in ref^27^, demonstrating its applicability to systems characterized by localized and homogeneous spatial regions partitioned by abrupt interfaces.

In this work, the inclusion of spherical MNPs in a PMMA medium doped with rare earth ions is studied, considering the UC process. The MNPBEM tool is used to obtain the conditions that result in maximum amplification of the electric field by the MNPs in the PMMA medium. The optimal size of the NPs and the excitation wavelength that maximizes the electric field intensity were determined. Subsequently, dopant ions are incorporated into the PMMA-NP system to investigate the upconversion process, the luminescent dynamics, and the ET process between donors and acceptors. After analyzing the luminescent dynamics, we identify the conditions that maximize the amplification of emission in the systems.

## Methods

Resonance ET (RET) occurs when a donor ion in the excited state transfers its energy to an acceptor ion in the ground state by a nonradiative process. The process does not involve the appearance of a photon. The distance at which RET is 50% efficient is called the Förster distance. The rate of Förster ET *W*_*ij*_ between the *i*-th donor and the *j*-th acceptor is defined by^28^
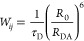
1where *R*_0_ represents the Förster distance, *R*_DA_ is the distance between the donor and acceptor, and τ_D_ is the fluorescence lifetime of the donor in the absence of the acceptor.

To simulate the luminescent dynamics of the system, consider *N*_D_ donors with one excited state and *N*_A_ acceptors with two excited states randomly distributed within a spherical volume. Let *P*_*i*_(*D*^0^) and *P*_*i*_(*D*^1^) be the probabilities that the *i*-th donor occupies the ground state and the excited state, respectively. Similarly, let *P*_*j*_(*A*^0^), *P*_*j*_(*A*^1^), and *P*_*j*_(*A*^2^) represent the probabilities that the *j*-th acceptor occupies the ground state, the first excited state, and the second excited state, respectively. The microscopic ratio equations for these two types of donors and acceptors, considering the UC-ESA and UC-ET, are the following
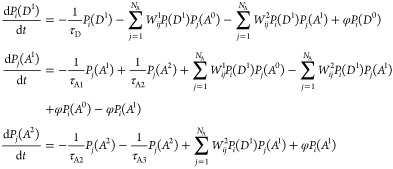
2where τ_A1_, τ_A2_, and τ_A3_ are the acceptor-free ion lifetimes for decays *A*^1^ → *A*^0^, *A*^2^ → *A*^1^, and *A*^2^ → *A*^0^, respectively. τ_D_ is the donor-free ion lifetime for *D*^1^ → *D*^0^, while *W*^1^ and *W*^2^ are the ET from the transitions (*D*^1^*A*^0^) → (*D*^0^*A*^1^) and (*D*^1^*A*^1^) → (*D*^0^*A*^2^), respectively. Finally, φ is the absorption pump rate for the transitions *D*^0^ → *D*^1^, *A*^0^ → *A*^1^, and *A*^1^ → *A*^2^ and is given by
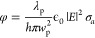
3where ϵ_0_ is the permittivity in vacuum, |*E*|^2^ is the electric field intensity, which is increased by surface plasmonic resonance of the MNP, λ_p_ is the pump wavelength, *w*_p_ is the pump radius, *h* is Planck’s constant, and σ_a_ is the absorption cross-section from the initial to the final energy level.^16^

The ET process described by the system of eq 2 unfolds as follows: first, the efficient interaction between incident light and silver or gold NPs excites the local enhanced electromagnetic field *E*. This excitation promotes certain donor ions from the ground-state *D*^0^ to an excited-state *D*^1^. The donor ions at this excited state can transfer their energy to the acceptor ions in the initial state *A*^0^ or *A*^1^ to a final energy state *A*^1^ or *A*^2^, respectively. The latter is through direct ET with a rate *W*_*ij*_. Alternatively, donor ions may dissipate their energy through multiphonon and photon relaxation processes with rates of 1/τ_D_. Once the acceptor ions are at excited states *A*^1^ and *A*^2^, they release energy through phonon and photon relaxation processes with a rate of 1/τ_1_ for transition *A*^1^ → *A*^0^, 1/τ_2_ for transition *A*^2^ → *A*^1^, and 1/τ_3_ for transition *A*^2^ → *A*^0^. The scheme of this process is shown in Figure 3a.

## Results and Discussion

By using the MNPBEM tool, the amplification of the electric field intensity was calculated, |*E*|^2^/|*E*_0_|^2^, as a function of the radius of the metallic nanosphere and the wavelength of the incident electromagnetic wave. |*E*|^2^ is calculated for a system consisting of a volume of PMMA with the MNP embedded in the center, while |*E*_0_|^2^ is calculated for an equal volume of PMMA without an MNP. The model developed in this work assumes a low concentration of MNPs in the PMMA matrix such that interactions between NPs, such as plasmonic coupling, are negligible. This approach ensures that the results obtained for a single NP are representative of the individual behavior, which remains valid in systems with sufficiently spaced NPs. PMMA was chosen as the matrix in this study due to its optical transparency in the studied range, as shown in previous studies,^29^ along with its chemical stability and ease of processing. These characteristics make it suitable for photonic applications and for studying plasmonic interactions in this spectral range. For calculations, a pulse of excitation of a plane wave with polarization along the *x* and *y* directions and a duration of 9τ_D_ was considered. To determine the total intensity of the electric field, a random distribution of 96,000 points in space was conducted and the electric field intensity at each point was calculated. Subsequently, the average of these magnitudes was computed, resulting in the value termed the intensity of the electric field (|*E*|^2^ or |*E*_0_|^2^).

**Figure 1:**
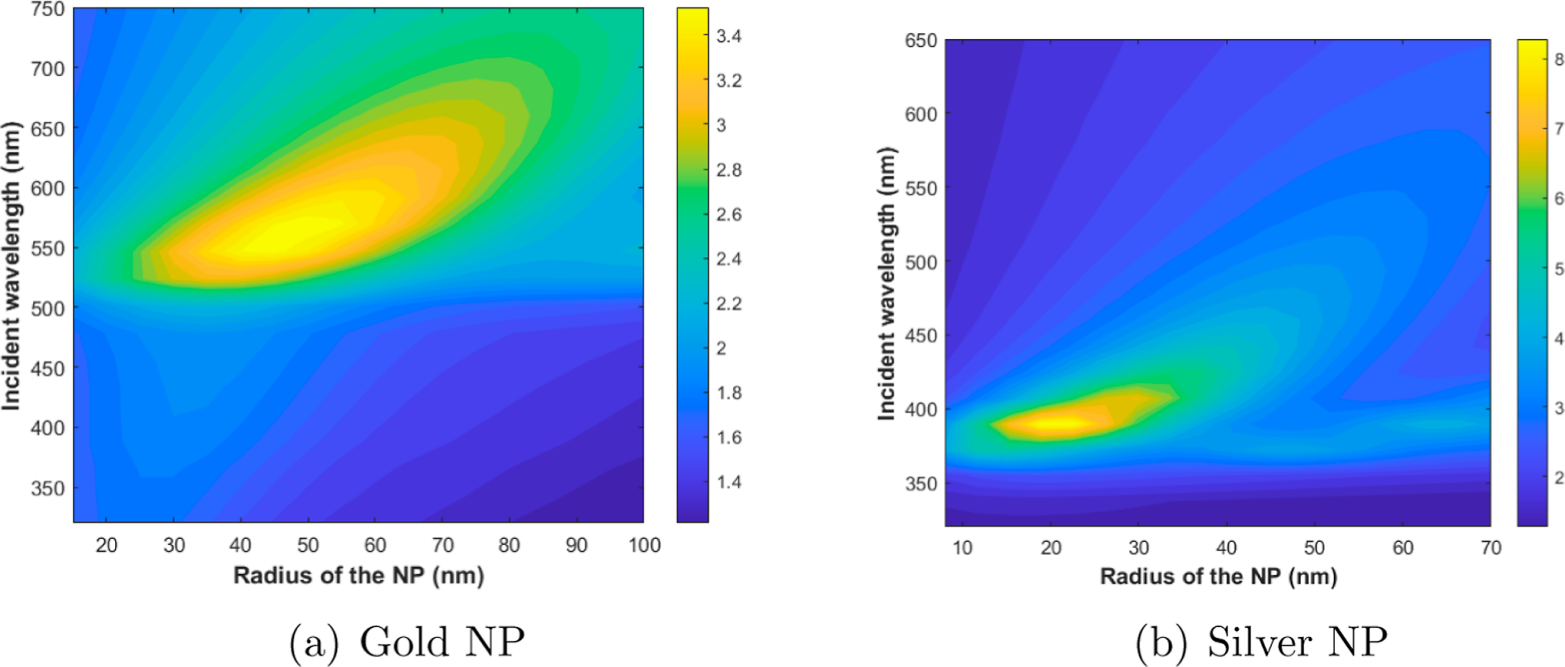
Amplification of the electric field intensity (|*E*|^2^/|*E*_0_|^2^) as a function of the radius of the NP (*r*_NP_) and the excitation wavelength (λ), for (a) a gold and (b) a silver NP.

Figure 1a depicts the plasmon amplification of the electric field intensity (|*E*|^2^/|*E*_0_|^2^) as a function of the radius of the gold NP within the range of 10–100 nm and the excitation wavelength from 320 to 750 nm. Similarly, Figure 1b presents the amplification of the electric field intensity for the silver NP for radii between 10 and 80 nm and an excitation wavelength range of 320 to 650 nm. In both figures, it can be clearly seen that there is a radius and an excitation wavelength for which the amplification of the electric field is the maximum. For the gold NP, the maximum is found for a radius close to 50 nm and an excitation wavelength of 568.9 nm, while for the silver NP, the optimal radius is around 20 nm and the wavelength excitation is 389.5 nm, which is in agreement with the data in the literature.^30,31^Table 1 shows the highest values of electric field amplification along with the radius of the MNPs and the incident wavelength that led to these maxima.

**Table 1:** Parameters That Maximize the Electric Field Intensity

metallic NP	radius of the NP (nm)	incident wavelength (nm)	|*E*|^2^/|*E*_0_|^2^
gold	50.0	568.9	3.6
silver	20.7	389.5	8.7

In both instances, electric field amplification is significantly notable, directly resulting from the incorporation of MNPs and the exploitation of their extraordinary plasmonic resonance properties. As a result, we achieve an amplification of the electric field by more than three times in the case of gold and by nearly nine times in the case of silver.

From Table 1, |*E*_Silver_|^2^/|*E*_Gold_|^2^ ∼ 2.4, that is, the silver NP amplifies the electric field more than twice as much as the gold NP. The higher amplification observed with silver NPs aligns with what the authors stated in ref^13^. This result is attributed to the higher extinction efficiency observed in silver NPs, as demonstrated in refs^13^ and^16^.

The geometry investigated here considers a single gold or silver nanosphere of radius *r*_NP_ embedded in a homogeneous PMMA medium. The dielectric constant values for PMMA are sourced from ref^32^ and^33^, while those for gold and silver in bulk are obtained from the experimental results of ref^34^. The simulations include NPs with a diameter less than 30 nm, for which it has been considered that the conduction electrons suffer additional damping due to surface dispersion or finite size confinement. Surface scattering effects depend on the size and shape of the particles, as discussed in ref^16^.

**Figure 2:**
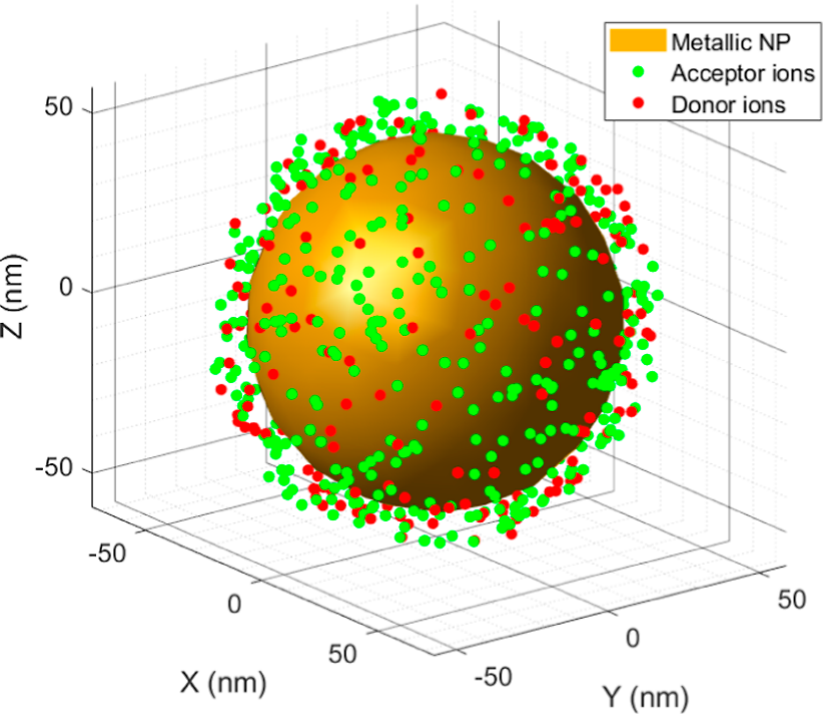
Example of a random distribution of ions around a MNP.

In the approach employed here, *N*_D_ donors and *N*_A_ acceptors are randomly distributed in a spherical shell around the MNP. This shell has an inner radius *r*_int_ = *r*_NP_ + 5 nm and outer radius *r*_ext_ = *r*_NP_ + 10 nm, illustrated in Figure 2. The model assumes that donors possess two energy levels (ground and excited states), whereas acceptors have three energy levels (ground and two excited states). The process includes excited state absorption (ESA) for both donor and acceptor ions as well as ET from donors to acceptors.

Once the optimal parameters for enhancing the electric field intensity amplification are determined, they are utilized to compute the emission from both donor and acceptor ions. This study incorporates variations in ion density while maintaining a fixed ratio between donors and acceptors, approximately . The total ion density *N*_T_ = *N*_A,T_ + *N*_D,T_ ranges from 1.15 × 10^18^ to 2.65 × 10^18^ ions/cm^3^. Here, *N*_A,T_ and *N*_D,T_ represent the total densities of acceptor and donor ions per cubic centimeter, respectively. In the proposed model for donors and acceptors, *N*_D,T_ = *N*_D_^0^ + *N*_D_^1^ and *N*_A,T_ = *N*_A_^0^ + *N*_A_^1^ + *N*_A_^2^, where the subscript indicates the type of ion (acceptor or donor), and the superscript denotes its energy level. The density of ions at each energy level is defined as follows
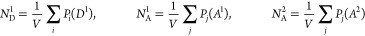
4

Here, *V* denotes the volume of the spherical shell housing the ions. The probabilities *P*_*i*_(*D*^1^) and *P*_*i*_(*A*^1,2^) are computed by using eq 2. As emission is directly proportional to the ion density at each energy level, the densities specified in eq 4 correspond to the emissions of donors and acceptors for each respective energy level.

**Figure 3:**
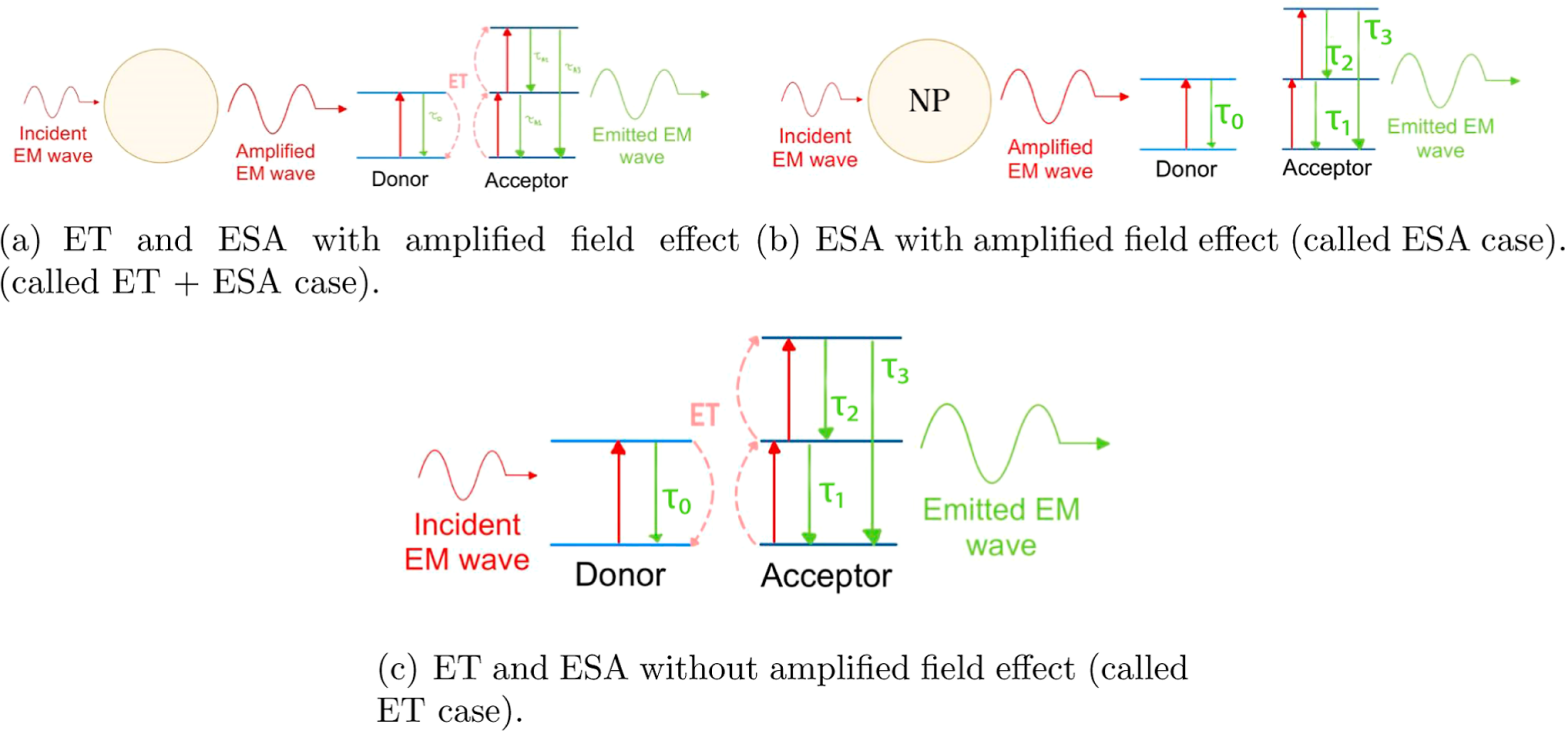
Schematic representation of the different emission scenarios considered here.

**Figure 4:**
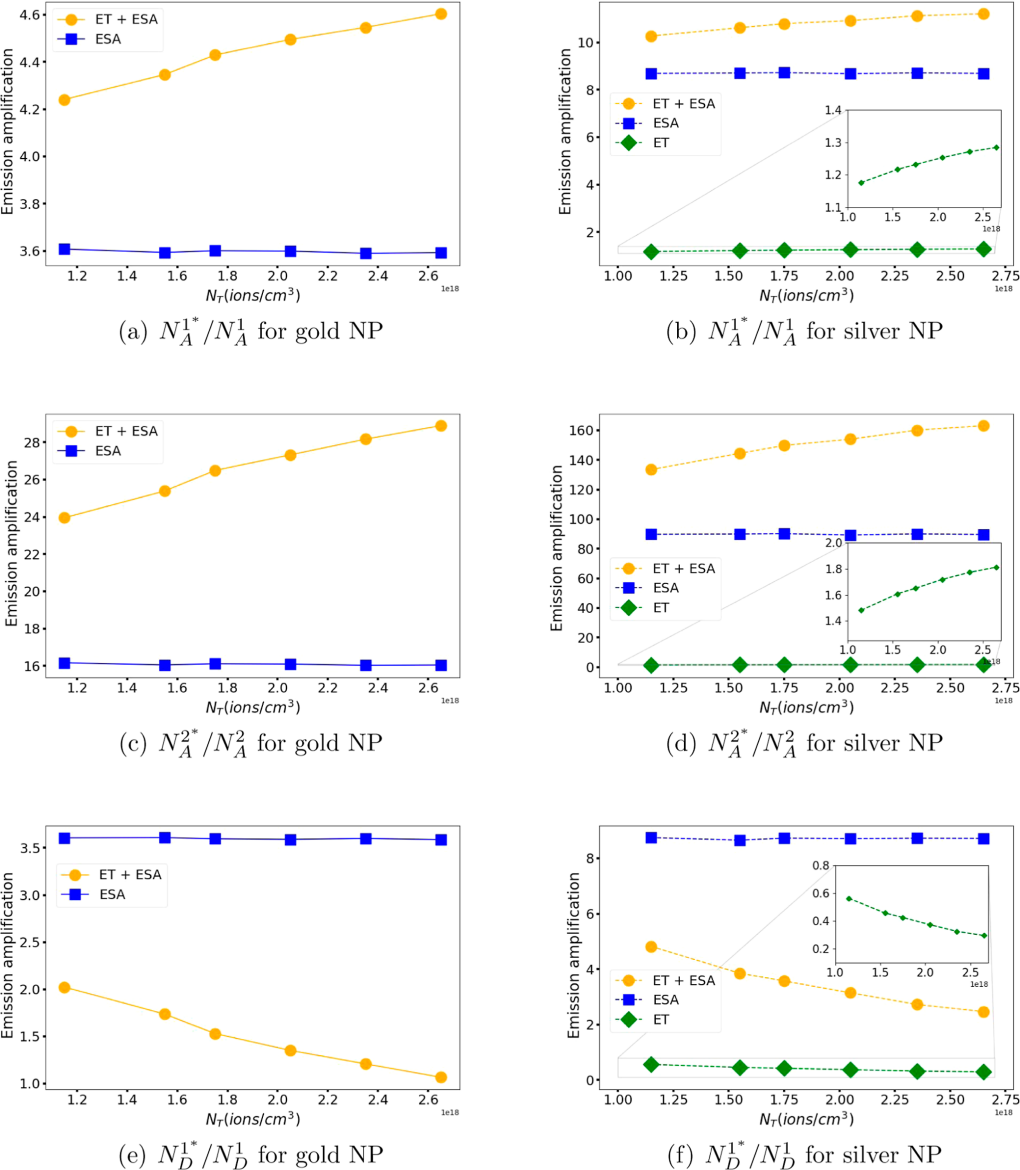
Amplification of the emission of (a,b) acceptors at level *A*^1^, (c,d) acceptors at level *A*^2^, and (e–f) donors at level *D*^1^ for metallic NPs. The figures on the left side (a,c,e) correspond to NP of gold (solid lines) and those on the right side (b,d,f) to NP of silver (dashed lines). The figures in the left column correspond to gold NP and those in the right column to silver NP. The ET + ESA (orange line •), ESA (blue line ■), and ET (green line ⧫) cases are shown for each energy level.

To demonstrate emission amplification, , , and  are analyzed. Here,  represents the acceptor emission at energy level 1 (level 2), while  is the donor emission at the excited level, under one of the scenarios shown in Figure 3. The first scenario incorporates the combined effects of excited state absorption (ESA) due to the amplified electric field and ET between ions (Figure 3a). The second scenario involves ESA induced solely by the amplified electric field (Figure 3b), while the last scenario (labeled as ET) considers ET and ESA without an amplified electric field (Figure 3c). In Figure 4, the emission amplification is plotted as a function of the total ion density for gold and silver NPs under the described scenarios. These calculations consider the optimal parameters previously identified: *r*_Gold_ = 50.0 *nm* and λ_Gold_ = 568.9 nm for gold NPs, and *r*_Silver_ = 20.7 nm and λ_Silver_ = 389.5 nm for silver NPs.

Figure 4a (4b), 4c (4d), and 4e (4f) shows the amplification of the emission of the ions at the excited levels *A*^1^, *A*^2^, and *D*^1^, respectively, for the system with a gold (silver) NP. The orange lines (•) correspond to the ET + ESA case (see Figure 3a), the blue lines (■) to the ESA case (see Figure 3b), and the green lines (⧫) correspond to the ET case (see Figure 3c). In general, similar behaviors are observed in the emission amplification of both acceptors and donors for both systems, whether they involve gold or silver NP. The most notable difference is in the amplitude of the amplification, which is observed to be greater in the case of the silver NP.

The ET case scenario is only shown in the figures corresponding to the silver NP; however, it should be emphasized that this scenario does not consider any NP. It is observed that the amplification of acceptor emission in the ET scenario (green dashed lines also shown in the inset in Figure 4b,d), that is, by ET from donor ions to acceptor ions increases as the density of the ions increases. This is because as the number of ions increases, the interactions between donors and acceptors also increase, which promotes ET. The energy fluctuation transfers from the donors to the acceptors, causing the acceptor ions to reach an excited state. This results in increased emission from the acceptors when there is greater donor–acceptor interaction. On the other hand, the donor emission amplification for the same scenario, shown in green dashed line in Figure 4f, decreases with increasing ion density. This behavior is due to the increase in donor–acceptor interactions as the number of ions increases, as previously observed. However, unlike the acceptors, the donor ions in an excited state transfer their energy to the acceptors instead of emitting it. Consequently, there are fewer donor ions available to emit energy.

In the ET scenario, in addition to ET, ground-state absorption due to pumping is also taken into account. The pumping field is uniform across the medium as there are no MNPs to create localized enhancements. Consequently, this uniform field excites more acceptors as their concentration increases, thereby contributing to observed emission amplification by increasing the number of optically active centers (acceptors).

In the ESA scenario (depicted by the blue lines in Figure 4), where the MNP enhances the electric field without considering ET, the amplification of donor and acceptor emissions remains consistent, despite variations in ion density. This stability arises because while emissions increase with additional ions in the presence of the MNP, they similarly increase even in its absence, thereby maintaining the unchanged correlation between the two emissions.

Finally, the amplification of the upconversion acceptor emission (second level) in the ET + ESA scenario (shown in orange lines in Figure 4a–d), which includes ET and the electric field enhancement by the MNP, increases as the ion density increases. This aligns with the observation that the interaction between donor and acceptor ET intensifies with an increase in ion density, as seen in the ET scenario. Additionally, the emission is further enhanced by the ESA effect, resulting in an amplification that exceeds the sum of both individual contributions. However, the amplification of donor emission in their first excited state in the ET + ESA scenario (shown in orange lines in Figure 4e,f) is lower than in the ESA scenario due to the presence of additional donor de-excitation channels influenced by the acceptors.

For analysis purposes, Table 2 displays specific values of emission amplification for each scenario corresponding to an ion density of 1.75 × 10^18^ ions/cm^3^. It can be seen that the amplification of acceptor emission at the first excited level (*N*_A_^1*^/*N*_A_^1^) for the ET + ESA scenario, where the electric field is enhanced by the presence of an MNP, is nearly equal to the sum of the effects observed individually for both gold and silver NPs. Specifically, for gold NP, 1.23_ET_ + 3.60_ESA_ = 4.83 compared with 4.43_ET+ESA_. Similarly, for silver NP, 1.23_ET_ + 8.76_ESA_ = 9.99 compared with 10.79_ET+ESA_.

**Table 2:** Acceptor and Donor Emission Amplification in Each Scenario for an Ion Density of 1.75 × 10^18^ ions/cm^3^

scenario	metallic NP	*N*_A_^1*^/*N*_A_^1^	*N*_A_^2*^/*N*_A_^2^	*N*_D_^1*^/*N*_D_^1^
ET + ESA	gold	4.43	26.48	1.53
	silver	10.79	149.83	3.57
ESA	gold	3.60	16.12	3.60
	silver	8.76	90.80	8.43
ET		1.23	1.64	0.43

However, for the second excited level of acceptors, the amplification emission (*N*_A_^2*^/*N*_A_^2^) exceeds the sum of the individual effects. More precisely, for gold NP, 1.64_ET_ + 16.12_ESA_ = 17.86 compared with 26.48_ET+ESA_. Correspondingly, for silver NP, 1.64_ET_ + 90.80_ESA_ = 92.44 compared with 149.83_ET+ESA_. This can be attributed to the fact that both effects (ESA and ET) interact with and enhance each other. The energy increases the number of excited states in the acceptors, making an ESA more likely. In turn, the ESA promotes ET by increasing the number of excited states in the acceptors. This mutual reinforcement forms a cycle that amplifies the overall signal beyond the additive effects of each process alone. This phenomenon underscores the importance of studying the ET process using upconversion techniques.

It is noteworthy that the emission amplification at the first excited level for both donors and acceptors in scenario ESA closely approaches the maximum electric field intensity amplification values (3.6 for gold NP and 8.7 for silver NP, as shown in Table 1). Conversely, the observed enhancement in emission at the second excited energy level of the acceptors in this scenario exceeds the expected increase based on the square of the electric field amplification, as predicted by the model presented in Appendix A. This discrepancy arises because the model in Appendix A, which assumes a homogeneous and infinite medium, does not fully align with the approach used here. This observation highlights the usefulness of our model as it accounts for varying ion distributions and the effect of the pump field intensity distribution in the medium, thereby providing a more accurate representation of the observed phenomena.

The last column of Table 2 shows that donor emission amplification in the ESA + ET scenario is the lowest. This aligns with the previous observation that ions excited by the ESA effect transfer their energy to the acceptor ions, resulting in fewer donor ions emitting their energy.

To compare our numerical results with experimental reports, we refer to the following references. Feng et al.^35^ reported that using silver nanowires significantly enhanced the upconversion emission of NaYF_4_:Yb, Er nanocrystals. According to their study, the intensities of the red and green upconversion emissions increased by factors of 3.7 and 2.3, respectively. In contrast, Zhan et al.^36^ fabricated gold nanorods with two distinct surface plasmon resonance peaks to simultaneously match the excitation and emission wavelengths of ZrO_2_:20%Yb^3+^, 2%Er^3+^@NaYF_4_:2%Yb^3+^ ultrasmall NPs (4 nm approx.) with spherical morphologies. Their study showed that the upconversion emission of the ZrO_2_ NPs was enhanced up to 35,000-fold only when the NPs are positioned at the tips of the gold nanorod, where the local electromagnetic field is strongest. Similarly, the study presented here demonstrates that the luminescent properties of PMMA doped with rare earth ions are significantly improved when MNPs, particularly silver and gold, are introduced. The enhanced electric field generated by surface plasmon resonance in these NPs amplifies the emission from rare earth ions. Specifically, silver NPs were found to enhance the electric field by up to 8.7 times, leading to a substantial emission amplification of approximately 150 times, far exceeding the performance of gold NPs, which enhanced the electric field by 3.6 times and led to an emission amplification of approximately 26 times. These findings are consistent with the experimental observations of enhanced luminescence due to plasmonic resonance reported by experimental studies.^23^

**Figure 5:**
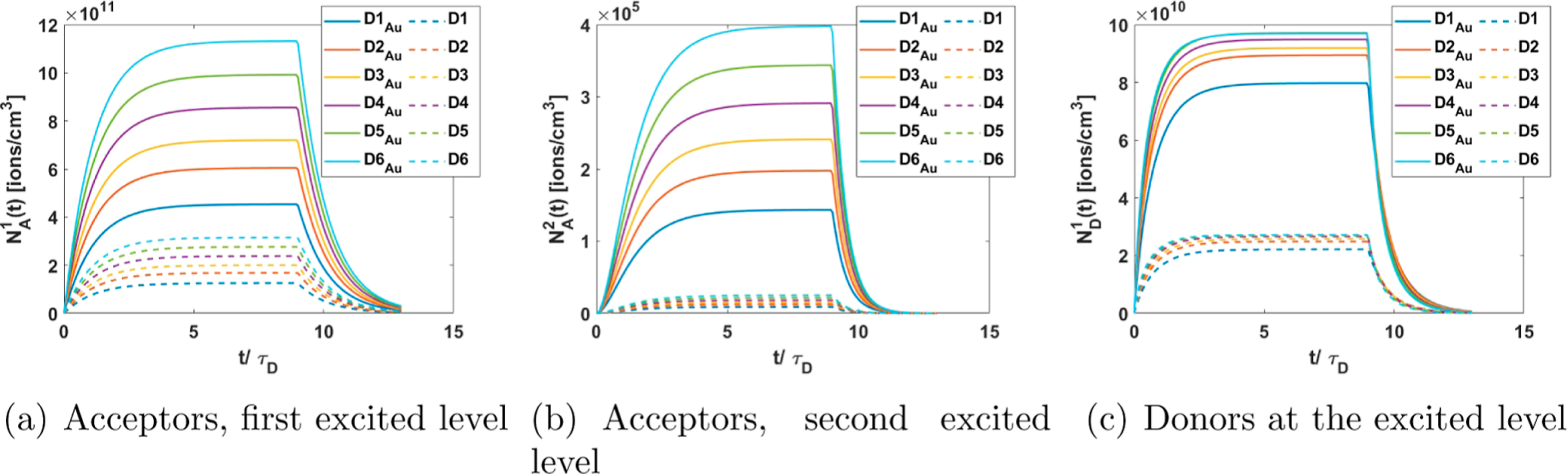
Decay curves for ions in the system with gold NP (solid lines) and for ions in the system without NP (dashed lines).

**Figure 6:**
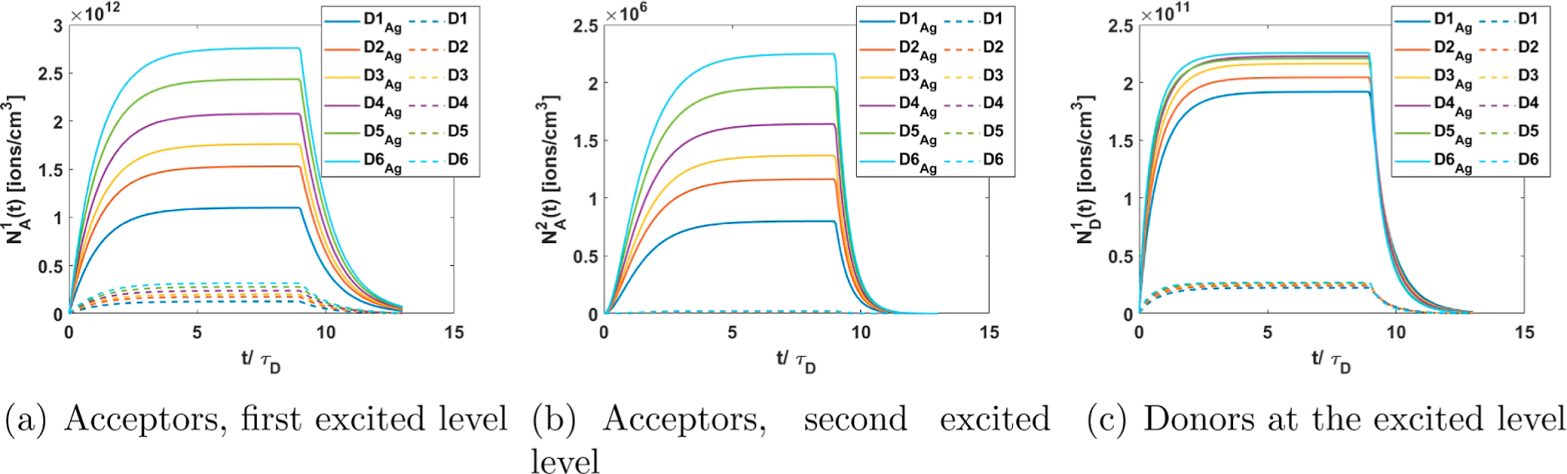
Decay curves for ions in the system with silver NP (solid lines) and for ions in the system without NP (dashed lines).

In addition to emission, the solutions of eq 2 also allow the calculation of the lifetimes of the donor and acceptor. Based on these solutions, decay curves are constructed for both ions, acceptors, and donors, in the ET and ET + ESA scenarios, shown in Figures 5 and 6 for gold and silver NP, respectively. To illustrate the effect of the ion density around the NPs, results are presented for various ion densities ranging from 1.15 × 10^18^ to 2.65 × 10^18^ ions/cm^3^, while maintaining a consistent acceptor-to-donor ratio of *N*_A,T_/*N*_D,T_ ∼ 2.5. The ion densities considered are *D*1 = 1.15 × 10^18^ ions/cm^3^, *D*2 = 1.55 × 10^18^ ions/cm^3^, *D*3 = 1.75 × 10^18^ ions/cm^3^, *D*4 = 2.05 × 10^18^ ions/cm^3^, *D*5 = 2.35 × 10^18^ ions/cm^3^, and *D*6 = 2.65 × 10^18^ ions/cm^3^. To demonstrate the effect of the MNP and provide a point of comparison, the results are displayed with solid lines for systems incorporating the MNP (ET + ESA scenario) and dashed lines for systems without the MNP (ET scenario), both under identical ion concentrations. These calculations consider a rectangular pulse whose duration is 9 times the free ion lifetime of the donors (τ_D_).

It is evident that the emission from both acceptors and donors (*N*_A_^1^(*t*), *N*_A_^2^(*t*), and *N*_D_^1^(*t*)) is significantly enhanced in the presence of metal NPs. This enhancement is evident from the comparison of the solid lines (representing conditions with MNP) and the dashed lines (without MNP) in Figures 5 and 6. Moreover, the emission from both acceptors (at their respective excited levels) and donors increases as the ion density increases. Additionally, systems incorporating silver NP (Figure 6) exhibit higher emission than those with gold NP (Figure 5).

It can be seen that as the excitation time progresses, the population densities increasingly approach their steady state. After the excitation period ends, the decay curves are recorded. The rise times reveal that donors reach their steady state rapidly (see Figures 5c and 6c), followed by acceptors in their first excited state (Figures 5a and 6a) and finally acceptors in their second excited state (Figures 5b and 6b).

**Figure 7:**
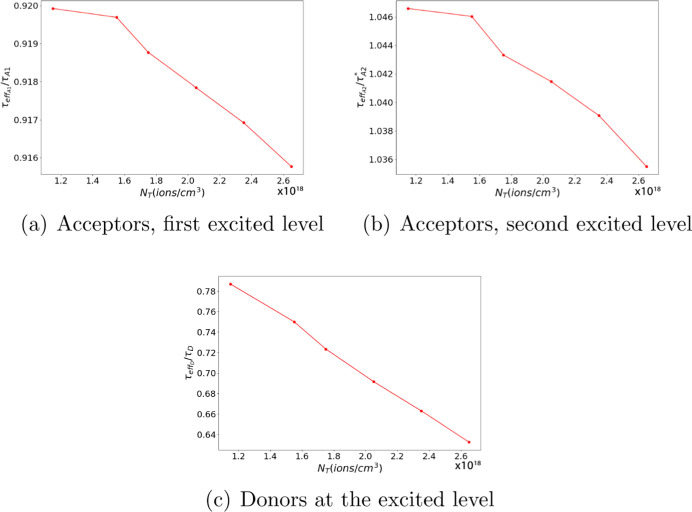
τ_effA1_/τ_A1_ as a function of ion density in the presence of a gold NP.

**Figure 8:**
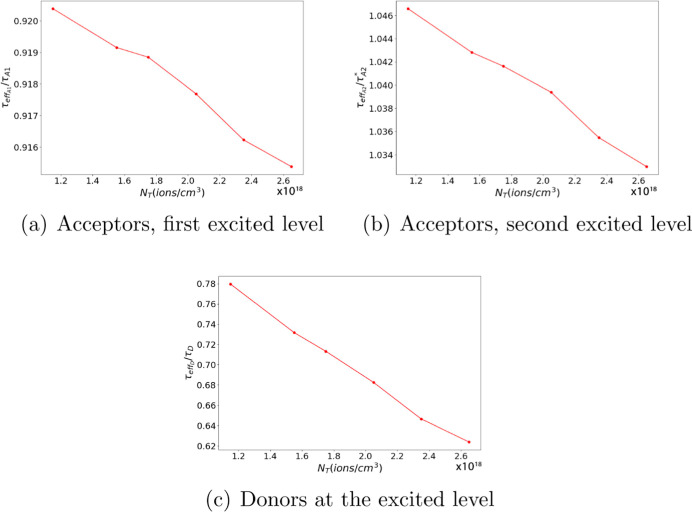
Effective lifetime as a function of ion density in the presence of a silver NP.

The effective lifetime of the ions, τ_eff_, is determined by fitting the decay curves, after emission begins to decline, using a single exponential function of the form exp(−*t*/τ_eff_). Figures 7 and 8 show the ratio of the effective lifetime in the ET + ESA case to the ion free lifetime as a function of ion density for the system with gold and silver NPs, respectively. The normalized values plotted in Figures 7 and 8 are , τ_effA1_/τ_A1_, and τ_effA2_/τ_A_2_^*^_, where τ_A_2_^*^_ = τ_A2_τ_A3_/(τ_A2_ + τ_A3_). These figures enable us to assess the impact of effective lifetime variations due to interactions between donor and acceptor ions by comparing these values to those observed without such interactions.

As expected, the effective lifetime of donors (Figures 7c and 8c) decreases with increasing total ion density as the donor–acceptor distance decreases, allowing more acceptors to receive energy from donors. Conversely, the decrease in the effective lifetime of acceptors with increasing total ion density (Figures 7a,b and 8a,b) is more complex but could be attributed to donors providing energy to excite acceptors. As donors decay faster due to ET to acceptors, fewer donors remain available to excite acceptors. It is noteworthy that the change in the effective lifetime of acceptors in their first and second excited states, respectively, is similar to the case in which donor and acceptor ions do not interact. In other words, in Figures 7 and 8, the ratio is close to one, although there is a slight decrease with increasing ion concentration.

The results presented here mark a significant progress in the comprehension and application of plasmonic resonance in MNPs, specifically gold and silver, for enhancing the luminescent properties of PMMA doped with rare earth ions. Our findings indicate that silver NPs, owing to their superior electric field enhancement capabilities, yield a more substantial amplification of luminescence compared to gold NPs. This enhancement is particularly notable in the second excited state of acceptor ions, underscoring the potential for significant emission enhancement through the meticulous optimization of plasmonic and doping conditions. The relationship between the ion density and emission amplification highlights the crucial role of ET and excited state absorption (ESA) processes in achieving optimal luminescent performance. Identifying NP sizes and excitation wavelengths that maximize the electric field intensity provides valuable insights for future material design and optimization.

The practical implications of this research are extensive, spanning from enhanced biomedical imaging and more sensitive biosensors to advancements in photovoltaic devices and other optical technologies. By harnessing the unique properties of rare earth-doped materials and leveraging the amplifying effects of plasmonic NPs, we can pave the way for developing next-generation luminescent materials with superior performance.

## Conclusions

This study investigates the luminescent dynamics of a PMMA-doped system with electric field enhancement via plasmonic resonance, facilitated by metallic spherical NPs. Förster’s theory and the MNPBEM toolbox were employed to calculate the luminescent dynamics. Optimal nanosphere radii for gold and silver were determined along with the excitation wavelength that maximizes electric field amplification around the NPs. The presence of metallic nanospheres enhances the electric field nearby, thereby amplifying the emission of doped ions through interaction. A nonspherical geometry may offer valuable insights into how shape influences electric field amplification, subsequently impacting the overall emission enhancement. This represents a potential avenue for future investigation. Importantly, previous studies did not establish a dependence of this amplification on ion density, a correlation revealed here by considering cumulative effects of ET and excited-state absorption (ESA) due to plasmonic resonance. Silver NPs exhibit over a 2-fold increase (∼2.4) in electric field intensity enhancement compared to gold NPs. Notably, significant emission amplification occurs at the second excited level of acceptor ions when NPs are present. Silver NPs, in particular, achieved a maximum emission amplification of approximately 150 times under optimal conditions. This finding underscores the potential of configurations that utilize upconversion processes to achieve substantially higher emission amplification compared to systems without upconversion processes under the same excitation conditions.

This study not only clarifies the mechanisms behind the enhanced luminescent properties of doped PMMA with MNPs but also lays the groundwork for future innovations and applications across various high-tech fields. We expect that the insights derived from this research will catalyze further exploration and advancement in the realm of advanced luminescent materials.

## Appendix A

Considering a macroscopic model with ions uniformly distributed in an infinite medium under homogeneous pumping intensity, the microscopic luminescent eq 2, without ET, has their macroscopic counterparts as follows

A.1

The solution of the steady state of the acceptor ions is as follows

A.2

where

A.3

and φ is the absorption pump rate, which is related to the electric field intensity through eq 3, φ ∝|*E*_0_|^2^.

Considering that , where *i* = 1, 2, and 3, we find that . Thus, the eqs (A.2) simplify as follows

A.4

Now, considering the amplification of the electric field, the emission in this case is given by

A.5

where φ* takes into account the amplification of the electric field (|*E*|^2^).

The previous equations allow for the calculation of emission amplification in a manner analogous to that used in Section 3, resulting in

A.6

Now, taking into account the expression (3), it is obtained that

A.7

From eq (A.7), it is evident that when ET does not influence the emission, the emission amplification of the first excited state is nearly proportional to the electric field intensity. Similarly, the emission amplification of the second excited state is nearly proportional to the square of the electric field intensity.
